# Sensitive, Fast, and Specific Immunoassays for Methyltestosterone Detection

**DOI:** 10.3390/s150510059

**Published:** 2015-04-29

**Authors:** Na Kong, Shanshan Song, Juan Peng, Liqiang Liu, Hua Kuang, Chuanlai Xu

**Affiliations:** State Key Lab of Food Science and Technology, School of Food Science and Technology, Jiangnan University, Wuxi 214122, China; E-Mails: kongxiyangsucc@126.com (N.K.); songshanshan0626@126.com (S.S.); pengjuan2016@163.com (J.P.); raxray@gmail.com (L.L.); kuangh@jiangnan.edu.cn (H.K.)

**Keywords:** icELISA, immunochromatographic strip assay, monoclonal antibody, methyltestosterone, fast detection

## Abstract

An indirect competitive enzyme-linked immunosorbent assay (icELISA) and an immunochromatographic strip assay using a highly specific monoclonal antibody, were developed to detect methyltestosterone (MT) residues in animal feed. The optimized icELISA had a half-inhibition concentration value of 0.26 ng/mL and a limit of detection value of 0.045 ng/mL. There was no cross-reactivity with eight analogues, revealing high specificity for MT. Based on icELISA results, the recovery rate of MT in animal feed was 82.4%–100.6%. The results were in accordance with those obtained by gas chromatography-mass spectrometry. The developed immunochromatographic strip assay, as the first report for MT detection, had a visual cut-off value of 1 ng/mL in PBS, 2.5 ng/g in fish feed, and 2.5 ng/g in pig feed. Therefore, these immunoassays are useful and fast tools for MT residue detection in animal feed.

## 1. Introduction

Synthetic androgens have been used in sports and stock farming since the 1950s due to their effects on muscle strength and animal meat production [1]. Since the late 1970s and 1980s, a large number of endocrine disorders linked to synthetic androgen residues in meat have been reported in humans [2,3]. Studies have shown that synthetic androgens negatively affect endocrine function in humans and animals [4,5], contributing to genital abnormalities, menstrual disorders, and sterility in humans, and imposex and reduced fertility in animals [6,7,8]. Accordingly, several countries, including China, have banned synthetic androgens in animal feed.

Methyltestosterone (MT), a synthetic androgen used in the treatment of infertility in humans, that increases weight gain and improves feeding efficiency in cattle [9]. However, MT in feed and its metabolite residues in meat can contribute to a series of adverse effects, therefore, several methods have been developed for the detection of MT residues. The most common detection methods are based on chromatography, including high performance liquid chromatography (HPLC) [10,11], liquid chromatography (LC) coupled with mass spectrometry (MS) [12], gas chromatography-mass spectrometry (GC-MS) [13,14] and LC-MS/MS [15,16]. These methods are both specific and sensitive, but they require tedious sample preparation, complicated spectral analyses, and trained operators, making these methods unsuitable for field detection. Compared with chromatography-based methods, immunoassays represent a rapid and simple method for the determination of MT. immunoassays include electrochemical immunosenors [17], chemiluminescent immunoassays [18,19], time-resolved fluoroimmunoassays [20] and enzyme-linked immunosorbent assay (ELISA). ELISA, which has both high selectivity and adequate sensitivity, have been increasingly used in sample analysis [21,22,23]. Furthermore, immunochromatographic strip assays represent alternative fast and simply methods.

In this study, we produced an anti-MT monoclonal antibody (mAb) and developed an indirect competive ELISA (icELISA) and immunochromatographic strip assay, which were applied to the rapid detection of MT residues in fish and pig feed samples.

## 2. Experimental

### 2.1. Reagents and Materials

MT (analytical standard) and relevant analogues, bovine serum albumin (BSA), ovalbumin (OVA), chlorauric acid (HAuCl_4_), carboxymethoxylamine hemihydrochloride (CMO, 98%), N-hydroxysuccinimide (NHS), 1-ethyl-3-(3-dimethylaminopropy) carbodiimide (EDC), Tween 20, gelatin, Freund’s complete adjuvant, Freund’s incomplete adjuvant, pancreatin, and 3,3',5,5'-tetramethylbenzidine (TMB) were purchased from Sigma-Aldrich (St. Louis, MO, USA). HRP-labelled goat anti-mouse IgG was supplied by Hua Mei Co (Shanghai, China). All reagents for cell fusion were obtained from Gibco BRL (Paisley, UK). All reagents were either analytical grade or HPLC-grade purchased from Beijing Chemical Co. (Beijing, China). Nitrocellulose (NC) membranes were purchased from Millipore Corporation (Bedford, MA, USA). Cellulose fiber, polystyrene backing card and glass fiber membranes were obtained from Goldbio Tech Co. Ltd. (Shanghai, China).

### 2.2. Instruments

The instruments in this study were a UV-Vis spectroscopy (Bokin Instruments, Tsushima, Japan), a microplate reader (MULTISKAN MKS, Thermo Labsystems Company, Philadelphia, PA, USA), a transmission electron microscope (JEOL, Tokyo, Japan), AirJet Quanti 3000™ and BioJet Quanti 3000™ units used as dispensers (Xinqidian Gene-Technology Co. Ltd., Beijing, China), and a strip cutter model CM 4000 (Gene, Shanghai, China). Water was purified using a Milli-Q Synthesis system (Millipore Co., Bedford, MA, USA).

### 2.3. Artificial Antigen Preparation

An MT derivative, MT-CMO, was synthesized by a modified oximation method [24]. The immunogen was prepared by conjugating MT-CMO to BSA using a modified 1-ethy-3-(3-dimethylaminopropy) carbodiimide (EDC) method [25]; the procedure is shown in Figure S1 (seeing Supplementary Materials). Briefly, 0.3 g of MT and 0.3 g of carboxymethoxylamine hemihydrochloride were mixed with 40 mL anhydrous pyridine in a dark chamber at 60 °C for 6 h. The residue was subjected to rotary evaporation and dissolved in 10 mL of methanol containing 10 mL of distilled water, and washed three times with 25 mL ethyl acetate. The organic phase was pooled and evaporated with anhydrous Na_2_SO_4_, resulting in MT-CMO, as a white solid.

In this experiment, 0.04 g of MT-CMO, 0.04 g of N-hydroxysuccinimide (NHS), and 0.06 g of EDC were dissolved in 3 mL anhydrous N,N-dimethylformamide (DMF) and stirred for 6 h at room temperature (RT) [26]. The mixture was added dropwise to 0.1 g of BSA dissolved in 12 mL of CBS (0.1 M, pH 9.6) and stirred for 12 h at RT. Following dialysis in PBS (0.01 M, pH 7.4) for 3 d (8 h/time), we obtained the immunogen MT-CMO-BSA. The conjugation between MT-CMO and BSA was assessed by UV-Vis spectroscopy. MT-CMO-OVA was prepared following the same procedures.

### 2.4. Preparation of Monoclonal Antibody (mAb) against MT

#### 2.4.1. Immunization of Mice

Female BALB/c mice (8–10 weeks old) were injected subcutaneously at multiple sites with the immunogen MT-CMO-BSA (100 μg in 0.1 mL saline) emulsified with an equal volume of Freund’s complete adjuvant. For subsequent boosters administered at one-month intervals, Freund’s incomplete adjuvant was used. Antisera were collected following the third booster immunization and screened for anti-MT activity by icELISA [27]. The mouse with the highest anti-MT activity was administered the immunogen (20 μg in 0.2 mL saline) by intraperitoneal injection and sacrificed three days later for cell fusion.

#### 2.4.2. Cell Fusion

The cell fusion procedures have been described elsewhere [28]. Firstly, SP2 cells were collected with pancreatin. Secondly, the spleen was removed in a sterile environment and ground to obtain the splenocytes. The splenocytes were washed several times with RPMI-1640 (Roswell Park Memorial Institute 1640). Thirdly, the splenocytes were fused with SP2 at a 10:1 ratio using PEG 1500 as the fusing agent. Following cell fusion, hybridomas were cultured with hypoxanthine aminopterin thymidine (HAT) medium and seeded on 96-well plates. Seven to ten days after fusion, hybridomas were screened by icELISA for the production of highly specific and sensitive anti-MT mAbs and cloned three times to obtain the hybridoma line by the limiting dilution method. Finally, the hybridoma line was expanded and cryopreserved.

#### 2.4.3. Production of Anti-MT mAb

Paraffin-primed BALB/c mice produced ascite fluids by injecting hybridoma cells into peritoneal cavity. The resulting mAbs were purified by saturated ammonium sulfate precipitation and stored at −20 °C in an equal volume of glycerin [29].

### 2.5. icELISA Procedure

**Figure 1:**
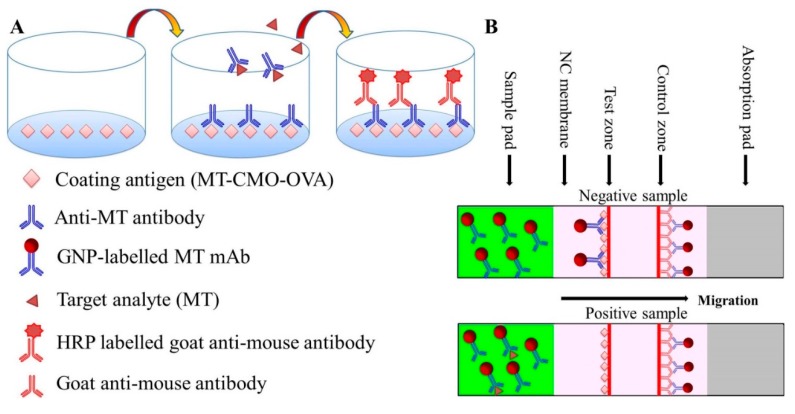
Schemes of icELISA (**A**) and immunochromatographic strip (**B**) for MT detection, respectively.

A schematic diagram of the icELISA procedure is shown in Figure 1. Checkerboard titration [30] was used for the determination of optimum coating antigen and antibody concentrations in icELISA. A 96-well microplate was coated with 100 μL/well MT-CMO-OVA (0.1 μg/mL, in 0.05 M CBS, pH 9.6) and incubated overnight at 4 °C. The wells were washed three times with washing buffer (0.01 M, PBS, containing 0.2% Tween 20) to remove free coating antigen and blocked with 220 μL blocking buffer (0.05 M, 0.2% Gelatin in CBS) at 37 °C for 2 h.

After washing, 50 μL of different concentrations of standard and 50 μL of antibody were added to wells and incubated at 37 °C for 30 min. Following another wash to remove unbound antibody and standards, 100 μL of 1:3000 diluted HRP-labelled goat anti-mouse IgG was added to each well and incubated for 30 min at 37 °C. After washing three times, 100 μL of substrate was added and incubated for 15 min at 37 °C. The enzymatic reaction was stopped with 50 μL sulfuric acid (2 M) per well. A microplate reader was used to determine the absorbance of each well at 450 nm.

### 2.6. icELISA Optimization

Three immunoassay parameters (methanol content, ionic strength and pH of the solvent) [31] were optimized to improve both the stability and sensitivity of icELISA. The criteria used to evaluate immunoassay performance were half inhibition concentration (IC_50_), maximum absorbance (A_max_) and A_max_/IC_50_. To optimize the methanol content in the solvent, PBS was mixed with different contents of methanol (0%, 5%, 10% and 20%). For ionic strength optimization, different concentrations of PBS (5, 10, 20, and 40 mM) were used. Finally, for solvent pH optimization, PBS was adjusted to different pH values (4.7–9.6).

### 2.7. Cross-Reactivity

In this study, mAb specificity was investigated by cross-reactivity (CR). Competition analogues (testosterone, nor-testosterone, estradiol, estriol, progesterone, epitestosterone, dihydrotestosterone and dexamethasone) were prepared in methanol at 1 mg/mL. A series of standard solutions (0, 1, 2, 5, 10, 20, 50 and 100 ng/mL) were diluted with optimized PBS and subjected to icELISA. CR values were calculated with the following formula:

CR (%) = (IC_50_ of MT/IC_50_ of competition analogue) × 100%

### 2.8. Gold Nanoparticle (GNP) Preparation

Gold nanoparticles (GNPs) with a mean diameter of 25 nm were prepared by the trisodium citrate reduction method [32]. All glassware used in the preparation of GNPs were soaked in *aqua regia* (HCl/HNO_3_ = 3:1, v/v), rinsed with ultrapure water several times, and air-dried. In this experiment, 100 mL of 0.01% HAuCl_4_ solution was heated to boiling and mixed with 2 mL of 1% sodium citrate solution under constant stirring. The color of the reaction solution changed from pale yellow to wine red within 1 min. The reaction solution was boiled for 15 min to complete the reduction of the HAuCl_4_, adjusted to 100 mL with ultrapure water, allowed to cool, and stored at RT. GNPs were characterized by UV-Vis spectroscopy at 200–800 nm and transmission electron microscopy [33].

### 2.9. Labelling of the MT mAb with GNPs

GNPs-labelled MT mAbs were prepared by a previously described method [34,35]. Under gentle and constant stirring, 10 mL of GNP solution was adjusted to pH 8.2 with K_2_CO_3_ (0.1 M). Subsequently, 100 μL of purified anti-MT mAb (1 mg/mL) diluted in borate buffer (0.1 M, pH 8.5) was added dropwise. Following incubation at RT for 1 h, 1 mL of 5% BSA was added slowly to stabilize the GNPs and block any residual surfaces on the GNPs [36]. Following a two-hour incubation, GNP-labelled MT mAbs were centrifuged at 8000 RPM for 12 min to remove the blocking agent and the excess antibody. The sediment was washed with gold-labelled re-suspension buffer [37] (10 mM PB, 5% sucrose, 1% BSA, 0.5% PEG 6000, 0.01% sodium azide, pH 7.2, w/v) and stored at 4 °C.

### 2.10. Immunochromatographic Strip Preparation

#### 2.10.1. Preparation of the Conjugate Pad

The conjugate pad was dispensed with the GNPs-labelled MT mAb on a glass fiber membrane using AirJet Quanti 3000™ and subsequently dried for 1 h at 37 °C. The pad was stored in a desiccator at RT.

#### 2.10.2. Immobilization of Capture Reagents

MT-CMO-OVA diluted to 1 mg/mL with CBS (0.01 M, pH 9.6) and goat anti-mouse IgG diluted to 0.5 mg/mL with PBS (0.01 M, pH 7.4) were applied to the test and control lines of the immunochromatographic strip. These capture reagents were sprayed onto the NC membrane with the BioJet Quanti 3000™. The sprayed width was 0.5 mm, and the sprayed volumes were 0.05 μL. After drying for 1 h at 37 °C, the NC membrane was stored in a desiccator at RT.

#### 2.10.3. Preparation of the Sample Pad and Absorbent Pad

In this experiment, 100% pure cellulose fiber was used for the sample and absorbent pads. Part of the cellulose fiber were saturated with PBS containing 0.2% Tween 20 and 1% BSA [38] as the sample pad and dried for 4 h at 37 °C. Another part of the cellulose fiber were used as the absorbent pad and stored in a desiccator at RT.

#### 2.10.4. Assembly of the Immunochromatographic Strip

A schematic representation of the immunochromatographic strip is shown in Figure 1. The immunochromatographic strip consists of three sections assembled in layers: three pads (sample, conjugate, and absorbent pad), a NC membrane, and a polystyrene backing card. The NC membrane with capture reagents was pasted on the central of the polystyrene backing card. The conjugate pad was attached on the polystyrene backing card with a 2-mm overlap on the NC membrane. The sample pad was pasted on the end justified to the conjugate pad, and the absorbent pad was pasted on the other side of polystyrene backing card with a 2-mm overlap on the NC membrane. Strips were sealed in a zip-lock bag, cut in 3-mm wide strips using a model CM 4000 strip cutter, and stored in a desiccator.

### 2.11. Test Procedure and Principle

MT standards of different concentrations (120 µL) were added onto the sample pad; the liquid migrated toward the absorbent pad. After 5 min, the results were observed. The color intensity of the test line is indicative of the amount of uncombined GNPs-labelled MT mAb. The higher the MT concentration in the sample, the lower the color intensity on the test line because MT prevents GNPs-labelled MT mAb from combining with MT-CMO-OVA. On the other hand, the lower the MT concentration in the sample, the higher the color intensity on the test line because GNPs-labelled MT mAb is trapped by MT-CMO-OVA. Therefore, there is a negative correlation between the color intensity of the test line and the concentration of MT in the sample.

### 2.12. Sample Analysis

#### 2.12.1. Sample Pretreatment

Fish and pig feed, which were obtained from the laboratory farm of our university, were confirmed to be MT-free by GC-MS. In this experiment, 2 g of finely ground pig and fish feed samples were transferred to 50 mL centrifuge tubes.

#### 2.12.2. For ELISA analysis

10 mL of acetonitrile aqueous solution (acetonitrile/H_2_O = 4:1) was added and extracted by ultrasonication for 20 min. The mixture was centrifuged at 4000 g for 5 min; 5 mL of the resulting supernatant was collected and dried by nitrogen. The residue was dissolved in 5 mL of methanol aqueous solution (methanol/H_2_O = 3:2) and mixed with 2 mL of ethyl acetate for defatting. The mixture was sonicated for 1 min, and the ethyl acetate was removed. The mixture was dried with nitrogen, and the residue was dissolved in 10 mL PBS (methanol/PBS = 1:9).

#### 2.12.3. For Immunochromatographic Strip Assay

10 mL of methanol aqueous solution (methanol/H_2_O = 2:3) was subjected to ultrasonication for 20 min. The mixture was centrifuged at 4000 g for 10 min, and the supernatant were pipetted onto the sample pad of immunochromatographic strip.

#### 2.12.4. Recovery

MT-negative fish and pig feed samples were spiked with three different concentrations of MT standard (1.25, 2.5, and 5 ng/g) and analyzed by GC-MS [39]. The samples were subsequently analyzed in triplicate by icELISA and the immunochromatographic strip assay.

## 3. Results and Discussion

### 3.1. Hapten Conjugation

MT-CMO was synthesized by oximation; its carbonyl group was converted into a carboxyl group. The carboxyl group of MT-CMO formed a link with the free amino groups present in BSA, thereby obtaining an immunogen (MT-CMO-BSA) (Figure S1, see Supplementary Materials). UV-Vis spectroscopy was used to determine whether the linking had been successful (Figure S2, see Supplementary Materials). MT-CMO-BSA had a characteristic absorption peak at 251 nm, which was similar to that of MT-CMO (255 nm), and had a novel absorption peak at 280 nm; therefore, the linking was successful. Based on the Lambert-Beer law [40] the conjugation ratio was 14 for MT-CMO-BSA. Similar results were obtained for the coating antigen (MT-CMO-OVA), which had a conjugation ratio of 9.

### 3.2. Production and Characterization of mAb

Serum was collected from mice following the third immunization. Mouse No. 16 had significant anti-MT activity based on icELISA results. Following two subsequent injections, the IC_50_ value was approximately 3 ng/mL, and the optical density (OD) was reduced from 1.4 to 0.6 with the addition of 3 ng/mL of MT in PBS. Therefore, mouse No. 16 was selected for cell fusion.

Hybridoma cells were cultured on 96-well plates. After seven days, the supernatants from wells were used for screening anti-MT antibodies. MT standards in PBS (0.5 ng/mL) were used to screen the sensitivity of the hybridoma cell line. Following subsequent cloning and screening, we obtained the hybridomas 1E12 cells, which were intraperitoneally injected into mice to produce ascites. Purified mAb had the IC_50_ value of 0.4 ng/mL, and it was increased approximately eight times in comparison to that of the antiserum.

### 3.3. Development and Optimization of icELISA

The optimal concentrations of the coating antigen (0.1 μg/mL) and anti-MT mAb (0.2 μg/mL) were determined by checkerboard titrations. Under these conditions, the standard curve was optimized. MT is a steroid that is soluble in methanol. Therefore, it is crucial to determine the methanol concentration in the icELISA solvent (*i.e.*, PBS). Increasing methanol concentrations increased Amax (Table S1, see Supplementary Materials), revealing that methanol has a promoting effect on the antigen-antibody reaction in icELISA. The optimum methanol concentration was 10% because Amax/IC_50_ had the highest value and IC_50_ had the lowest value. The result was also used to redissolve MT residue from the real sample.

Increasing the concentration of PBS from 5 to 40 mM decreased Amax due to the PBS concentration increasing to cause the enhancement of ionic strength (Table S1, see Supplementary Materials). Ions bind to paratopes or charged groups of epitopes, inhibiting antigen-antibody reaction, and inducing decreased Amax [41]. Therefore, the optimum ionic strength of PBS was 10 mM.

Five pH values (4.7, 6.0, 7.4, 8.6, and 9.6) were evaluated. The icELISA was sensitive at neutral (pH 7.4) and acidic conditions (pH 4.7) with IC_50_ values of 0.455 and 0.456 ng/mL, respectively (Table S1, see Supplementary Materials). However, low pH values contributed to low Amax and this phenomenon was attributed to the low reaction activities between antigen and antibody. Therefore, pH 7.4 was selected as the optimum pH value for icELISA.

**Figure 2:**
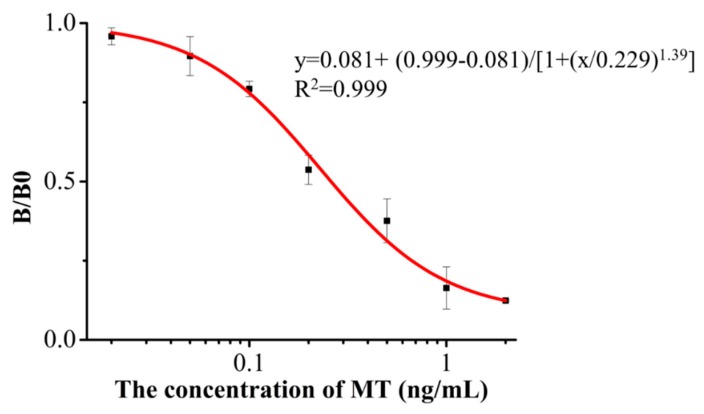
Optimized inhibition standard curve for MT analysis by icELISA. Error bar was calculated according to three repeats at each concentration.

In conclusion, the MT standard was diluted with PBS (10 mM, pH 7.4) and analyzed by icELISA. The standard curve was established by plotting the B/B0 (B was the absorbance at each concentration of MT and B0 was the absorbance in the absence of MT) against the concentration of MT (Figure 2). The standard curve equation was *y* = 0.081 + (0.999 − 0.081)/(1 + (*x*/0.229)^1.39^), the linear regression correlation coefficient (*R^2^*) was 0.999, the IC_50_ value was 0.26 ng/mL, and the limit of detection (LOD, concentration calculated as IC_10_) was 0.045 ng/mL.

### 3.4. Specificity of mAb

The specificity of mAb was assessed by CR. Eight of analogues were analyzed by icELISA (Table 1). Testosterone had a CR rate of approximately 2.17%, because testosterone without methyl group at the C-17 positon. The CR rate with nor-testosterone (1.04%) and estradiol (0.52%) was calculated. There were two extra methyl groups located in the C-17 and C-18 positions of these compounds, which were not present in MT. The results agreed with the immunogen synthesis scheme. Even though these analogues have CR with mAb, the rates were <2.5% [29]. There was no CR with other analogues because the changed carbonyl group and the extra fluorine atom have significant effects on affinity. Therefore, the results revealed that the mAb had high specificity against MT.

**Table 1:** The cross-reactivity of anti-MT mAb with analogs.

Analogs	IC_50_ (ng/mL)	CR (%)
Methyltestosterone	0.26	100
Testosterone	12	2.17
Nor-testosterone	25	1.04
Estradiol	50	0.52
Estriol	>100	<0.26
Progesterone	>100	<0.26
Epitestosterone	>100	<0.26
Dihydrotestosterone	>100	<0.26
Dexamethasone	>100	<0.26

### 3.5. Analytical Characteristics of the Immunochromatographic Strip

**Figure 3:**
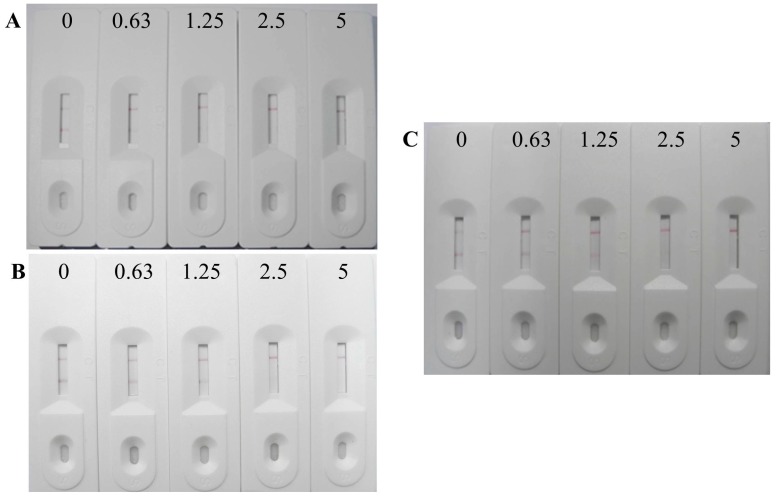
Photograph of the immunochromatographic strip for MT detection in PBS (**A**); fish feed (**B**) and pig feed (**C**). The concentrations of MT from left to right were 0, 0.63, 1.25, 2.5, and 5 ng/g.

The MT stock solution was diluted to different concentrations (0, 0.63, 1.25, 2.5 and 5 ng/mL) with PBS and loaded onto the sample pad. After 5 min, the results were obtained (Figure 3). At 0.63 ng/mL MT, the color intensity of test line was lower than of the control line. At 1.25 ng/mL MT, no test line was observed. Therefore, the visual cut-off value of the immunochromatographic strip assay was 1.25 ng/mL.

### 3.6. Sample Matrix Effects on the Immunochromatographic Strip Assay

To determine if the immunochromatographic strip assay whether could be used for detection of MT in real samples (fish and pig feed), these samples spiked with different concentrations (0, 0.63, 1.25, 2.5 and 5 ng/g) of MT. As shown in Figure 3, the test line was visible at MT < 1.25 ng/g; however, that line disappeared at 2.5 ng/g MT. Owing to the complex compositions in the real samples rather than the straightforward compositions in PBS, the visual cut-off values in real samples were higher than that in PBS. The test was repeated 20 times using different batches of animal feed. The consistency in the results confirmed that the immunochromatographic strip assay is highly reproducible.

### 3.7. Comparisons among icELISA, Immunochromatographic Strip Assay and GC-MS

**Figure 4:**
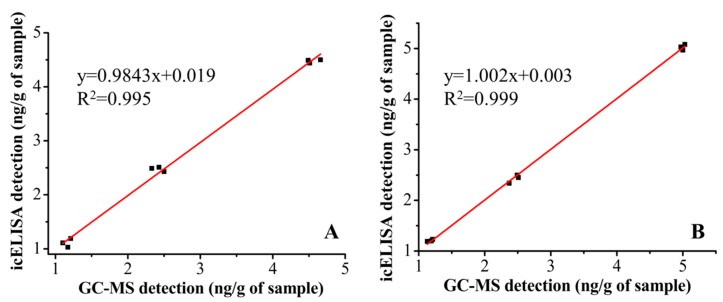
Correlation studies between icELISA and GC-MS detection in fish (**A**) and pig (**B**) feed. These samples were obtained from MT spiked at levels of 1.25, 2.5 and 5 ng/g (*n* = 3).

The results of this experiment are shown in Figure 4. Samples were spiked with MT (1.25, 2.5 and 5 ng/g), with three replicates per concentration. The linear regression equations between the icELISA and GC-MS methods was *y* = 0.9843*x* + 0.019 (fish feed) and *y* = 1.002*x* + 0.003 (pig feed). The two methods were highly correlated with *R^2^* values of 0.995 and 0.999 for fish feed and pig feed samples, respectively. To our knowledge, the sensitivity and specificity of the icELISA were superior to those of previous published reports. The comparisons were summarized in Table S2 (see Supplementary Materials) [42,43,44]. Comparisons between icELISA, the immunochromatographic strip assay, and GC-MS were performed (Table 2). The results obtained from icELISA and the immunochromatographic strip assay were consistent and sensitive for detection of MT in real-samples. Additionally, icELISA and the immunochromatographic strip assay do not have the limitations of GC-MS, *i.e.*, expensive equipment and tedious sample preparation. Furthermore, the results were judged within 5–10 min by the immunochromatographic strip assay, making it a powerful tool for field analyses.

**Table 2:** Comparison of MT analyses by icELISA, GC-MS and immunochromatographic strip in animal feed (*n* = 3).

		icELISA		GC-MS		Test Strips
Samples	Spiked (ng/g)	Detected (ng/g), Mean ± SD	Recovery (%)	Detected (ng/g), Mean ± SD	Recovery (%)	Detected ^a^
Fish feed	0	ND	NC ^b^	ND	NC ^b^	---
	1.25	1.03 ± 0.07	82.4	1.17 ± 0.13	93.6	±±±
	2.5	2.51 ± 0.12	100.4	2.43 ± 0.02	97.2	+++
	5	4.49 ± 0.22	89.8	4.51 ± 0.09	90.2	+++
Pig feed	0	ND	NC ^b^	ND	NC ^b^	---
	1.25	1.19 ± 0.12	95.2	1.21 ± 0.23	96.8	±±±
	2.5	2.34 ± 0.08	93.6	2.51 ± 0.09	100.4	+++
	5	5.03 ± 0.15	100.6	4.97 ± 0.14	99.4	+++

^a^ -, absence of MT; +, presence of MT; ±, weakly positive; ^b^ not calculated.

## 4. Conclusions

In this study, a mAb against MT was produced and characterized. An icELISA and an immunochromatographic strip assay were developed for detection of MT residue in animal feed. To our knowledge, the sensitivity and specificity of the icELISA are better than those of previously published reports. Furthermore, it is the first time an immunochromatographic strip assay developed for MT residue analysis in animal feed is reported. Those immunoassays were sufficiently sensitive and accurate for the rapid analysis of MT.

## Supplementary Files

Supplementary File 1
