# Supplementary material for: Sensitive, Fast, and Specific Immunoassays for Methyltestosterone Detection
Source: Sensors (Basel). 2015 Apr 29;15(5):10059–73. doi: 10.3390/s150510059 (PMC4481955; doi:10.3390/s150510059)
Supplement: Supplementary File 1 [file sensors-15-10059-s001.pdf]

OPEN ACCESS

sensors

ISSN 1424-8220

www.mdpi.com/journal/sensors

Supplementary Information

Sensitive, Fast, and Specific Immunoassays for Methyltestosterone Detection. *Sensors* 2015, *15*, 10059-10073

Na Kong, Shanshan Song, Juan Peng, Liqiang Liu, Hua Kuang and Chuanlai Xu *

State Key Lab of Food Science and Technology, School of Food Science and Technology, Jiangnan University, Wuxi 214122, Jiang Su, China; E-Mails: kongxiyangsucc@126.com (N.K.); songshanshan0626@126.com (S.S.); pengjuan2016@163.com (J.P.); raxray@gmail.com (L.L.); kuangh@jiangnan.edu.cn (H.K.)

* Author to whom correspondence should be addressed; E-Mail: xcl@jiangnan.edu.cn; Tel.: +86-510-8532-9076.

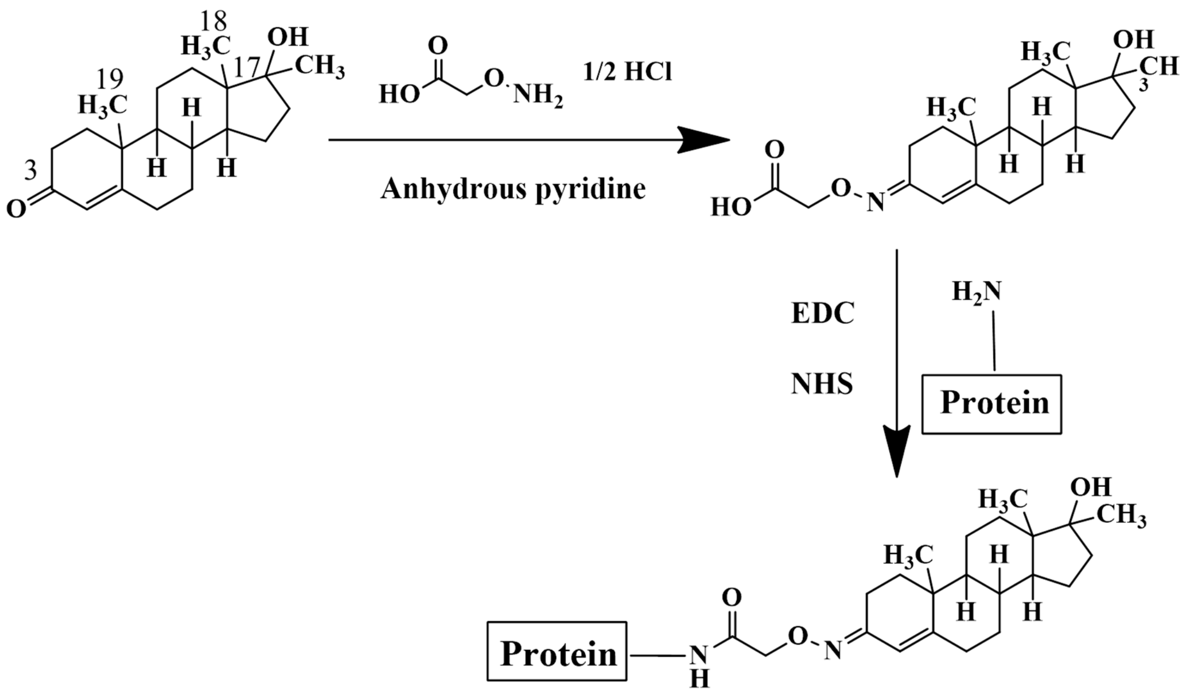

Figure S1. The synthesis routes to MT-CMO and MT artificial antigen by the EDC method. The labels 3, 17, 18 and 19 correspond to the carbon bond positions mentioned in the article.

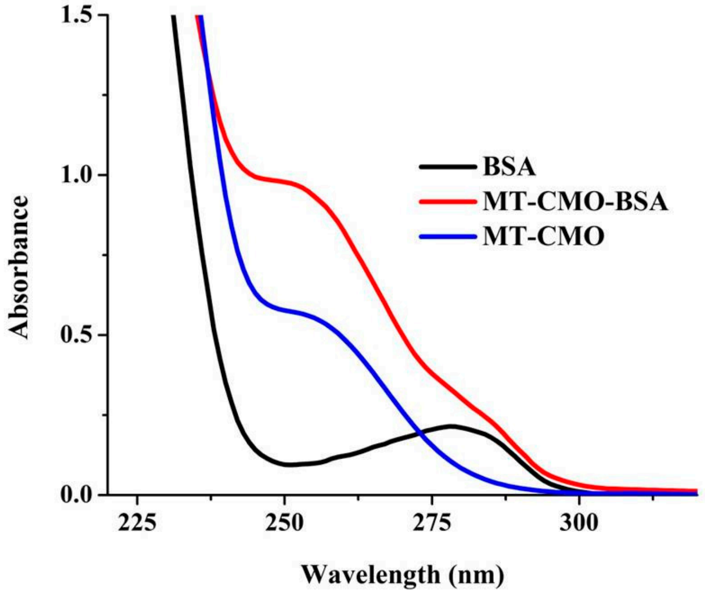

Figure S2. UV-Vis spectra of the immunogen for MT.

Table S1. Effect factors of the optimized icELISA.

**Factor**	$\mathbf{A_{max}}$	$\mathbf{IC_{50}}$	$\mathbf{A_{max}/IC_{50}}$
		Methanol Content (%)	
0	1.478 ± 0.015	0.418 ± 0.02	3.536 ± 0.032
5	1.577 ± 0.026	0.567 ± 0.023	2.783 ± 0.009
10	1.704 ± 0.011	0.418 ± 0.05	4.077 ± 0.05
20	1.873 ± 0.049	0.626 ± 0.009	2.992 ± 0.004
		Ionic strength [a] (mM)	
5	1.632 ± 0.034	0.68 ± 0.017	2.400 ± 0.05
10	1.578 ± 0.023	0.398 ± 0.024	3.965 ± 0.013
20	1.164 ± 0.022	0.565 ± 0.024	2.060 ± 0.07
40	0.895 ± 0.036	0.504 ± 0.092	1.776 ± 0.043
		pH	
4.7	0.850 ± 0.002	0.456 ± 0.005	1.864 ± 0.003
6.0	1.741 ± 0.005	0.723 ± 0.038	2.408 ± 0.134
7.4	1.428 ± 0.025	0.455 ±0.029	3.138 ± 0.063
8.6	1.686 ± 0.051	0.577 ± 0.026	2.922 ± 0.023
9.6	1.490 ± 0.045	0.714 ± 0.007	2.087 ± 0.045

[a] the concentration of PBS.

Table S2. Comparison of LOD and CR for MT detection based on ELISA.

CR (%)	LOD (ng/mL)	Ref.
No mention	0.141	42
<78	0.266	43
non-specificity	200	44
<2.17	0.045	This work

© 2015 by the authors; licensee MDPI, Basel, Switzerland. This article is an open access article distributed under the terms and conditions of the Creative Commons Attribution license (http://creativecommons.org/licenses/by/4.0/).
